# *In Vitro* Large Scale Production of Human Mature Red Blood Cells from Hematopoietic Stem Cells by Coculturing with Human Fetal Liver Stromal Cells

**DOI:** 10.1155/2013/807863

**Published:** 2013-01-30

**Authors:** Jiafei Xi, Yanhua Li, Ruoyong Wang, Yunfang Wang, Xue Nan, Lijuan He, Peng Zhang, Lin Chen, Wen Yue, Xuetao Pei

**Affiliations:** Stem Cell and Regenerative Medicine Lab, Beijing Institute of Transfusion Medicine, 27 Taiping Road, Beijing 100850, China

## Abstract

*In vitro* models of human erythropoiesis are useful in studying the mechanisms of erythroid differentiation in normal and pathological conditions. Here we describe an erythroid liquid culture system starting from cord blood derived hematopoietic stem cells (HSCs). HSCs were cultured for more than 50 days in erythroid differentiation conditions and resulted in a more than 10^9^-fold expansion within 50 days under optimal conditions. Homogeneous erythroid cells were characterized by cell morphology, flow cytometry, and hematopoietic colony assays. Furthermore, terminal erythroid maturation was improved by cosculturing with human fetal liver stromal cells. Cocultured erythroid cells underwent multiple maturation events, including decrease in size, increase in glycophorin A expression, and nuclear condensation. This process resulted in extrusion of the pycnotic nuclei in up to 80% of the cells. Importantly, they possessed the capacity to express the adult definitive **β**-globin chain upon further maturation. We also show that the oxygen equilibrium curves of the cord blood-differentiated red blood cells (RBCs) are comparable to normal RBCs. The large number and purity of erythroid cells and RBCs produced from cord blood make this method useful for fundamental research in erythroid development, and they also provide a basis for future production of available RBCs for transfusion.

## 1. Introduction

Erythropoiesis defines the process of differentiation and proliferation from hematopoietic stem cells (HSCs) to mature red blood cells (RBCs). In adult humans, erythroid differentiation produces about 2 × 10^11^ red cells per day. Erythroid lineage development requires a delicate balance between the opposing effects of proliferation promoting factors and differentiation-inducing factors. The two most important cytokines are erythropoietin (EPO) and stem cell factor (SCF) [1]. EPO protects erythroid progenitor cells from apoptosis by activating antiapoptotic proteins, and it also stimulates hemoglobin (Hb) synthesis and is essential for terminal differentiation [2]. SCF acts synergistically with this lineage-specific factor by promoting proliferation of erythroid progenitor cells [1]. A variety of other factors, such as insulin-like growth factor 1 (IGF-1), insulin, and glucocorticoids, have been proposed to have a supportive effect on human RBCs development. In this way, IGF-1 enhances proliferation as well as terminal differentiation, by which it promotes nucleus condensation and enucleation [3]. Glucocorticoids, such as dexamethasone (Dex), seem to have a direct influence on the proliferation of erythroid progenitor cells [4]. *In vivo*, both renewal and maturation of human erythroid progenitors proceed in parallel in the bone marrow. It thus has been difficult to assess the contribution of particular signaling pathways and their deregulation during erythroid cell development.

Despite this knowledge about RBC development, most of the mechanisms underlying erythroid differentiation and maturation both at cellular and molecular levels still remain unknown. In particular, the mechanisms of enucleation, a unique phenomenon of human erythropoiesis, are still unknown. Thus, the availability of an *in vitro* erythropoiesis model, which exhibits all stages of RBC development, would be a powerful tool for investigating the factors and molecular mechanisms involved in proliferation and differentiation of human erythroid cells under normal and pathophysiologic conditions. Now, most information was obtained from established cell lines and primary animal cell models of chicken and mouse in defined media [2, 5]. 

Most of the commonly available *in vitro* assays of erythropoiesis are based on cell lines, such as murine MEL or human K562, HEL, and UT-7 cell lines. However, cell lines usually do not recapitulate the entire process of erythropoiesis, as many regulatory pathways have been altered during the transformation process that led to their immortalization. Therefore, for a long time, efforts have been made to establish* in vitro* unilineage differentiation of human primary erythroid cells. 

Cord blood has received a great deal of attention as a source for HSCs as an alternative to bone marrow stem cells in transplantation medicine [6, 7]. Furthermore, cord blood is highly enriched in committed hematopoietic progenitors, including those of the erythroid lineage [8]. We addressed this problem by *in vitro* large scale production of erythroid cells from umbilical cord blood derived HSCs.

Decades ago, most *in vitro* experiments were performed under semisolid culture conditions. This approach had the disadvantage that only immature differentiation stages were generated and the relatively small cell numbers derived in colonies severely limited subsequent investigations. In addition, the options for investigating the influence of specific growth hormones were restricted. To overcome these limitations, liquid cultures (LCs) have been developed over the past few years. Most LCs, however, showed only moderate proliferation or an absence of terminal differentiation and enucleation [9–11]. These methods are difficult to test the effects of various factors at different maturation stages since it is difficult to add or subtract components to/from the culture. However, the use of liquid cultures is also limited either by the production of mixed erythroid and myeloid cells or by a weak or absent terminal enucleation [12].

Several reports suggest that hematopoietic niche cells may promote the terminal enucleation of erythroid cells *in vitro* [13, 14]. Recently, Isern, et al. report that the fetal liver (FL) provides a previously unrecognized developmental niche for the maturation and enucleation of primitive erythroid cells (EryP). These results demonstrate that the FL is a niche for maturation of primitive erythroid cells [15]. And the FL is also a major site for the development of definitive erythroid cells (EryD), which matures within erythroblastic islands (EBIs) [16]. EBIs, first identified in bone marrow and later in FL and spleen, are morphologically distinct 3D structures comprising a central macrophage surrounded by EryD at various stages of maturation [17]. Studies have revealed macrophage extensions that surround peripheral erythroblasts, providing intimate membrane contact between these cells. The central macrophages of the EBIs are thought to function as nurse cells during erythropoiesis [17–19]. Therefore, we hypothesized that unknown growth proteins are produced as yet unidentified populations of fetal liver stromal cells that stimulate the maturation of erythroid progenitor cells.

Here, we thoroughly describe optimized long-term serum-free culture conditions that allowed expansion into mass cultures of highly homogeneous human erythroid progenitors derived from umbilical cord blood. Cells could be routinely expanded for more than 50 days, undergoing up to 35 population doublings. The mature red blood cells could be produced by coculturing erythroid progenitors with human fetal liver stromal cells (hFLSCs). With this procedure, it is possible to reproduce different steps of human normal erythropoiesis. The large number and purity of erythroid cells produced from a small amount of peripheral blood make this method useful for studying either normal or pathological erythropoiesis. In addition, production of mature red blood cells from cord blood derived HSCs is of great potential and of importance in practice for it could eventually become an alternate source of cell for transfusion.

## 2. Materials and Methods

### 2.1. *In Vitro* Culture and Characterization of Erythroid Cells from Cord Blood HSCs

After informed consent had been obtained, cord blood from healthy volunteers was collected into sterile heparinized tubes. Mononuclear cells were obtained by centrifugation on Lymphoprep density gradient. The mononuclear cells were then enriched for HSCs by positive selection using anti-CD34-tagged magnetic beads using Mini-MACS columns (Miltenyi Biotech, Auburn, CA) according to the manufacturer's protocol. Purity was more than 95%; as determined by flow cytometry. CD34 positive cells were cultured as described previously [20, 21]. Briefly, for initial expansion, 1 × 10^6^ cells/mL were cultivated in serum-free medium (StemSpan; Stem Cell Technologies, Vancouver, BC, Canada) supplemented with Epo (2 U/mL; Sigma-Aldrich, St. Louis, MO), the synthetic glucocorticoid Dex (1 *μ*M; Sigma), IGF-1 (40 ng/mL; R&D Systems, Minneapolis, MN), SCF (100 ng/mL; R&D Systems), and iron-saturated human transferrin (400 *μ*mg/mL; Sigma-Aldrich). 

Homogeneous cultures of erythroid progenitors established after several days were kept in the same medium at 2 × 10^6^ cells/mL by daily partial medium changes. As the cultured erythroblasts being over 5 × 10^7^ (about 20 wells of 24-well plate), we purified the erythroblasts by density centrifugation (Ficoll, 1.077 g/mL). And then only about 2 × 10^6^ cells (about 20 wells of 24-well plate) were continued to be cultured. And the cells lost by density centrifugation were corrected when calculating the proliferation of erythroid cells. Proliferation kinetics of cells were daily monitored using an electronic cell counter (Vi-cell XR, Beckman Coulter, Germany); cumulative cell numbers were calculated as previously described [22]. Cumulative cell numbers were calculated and plotted against time.

Cell morphology was analyzed by light microscopy on cytocentrifuged smears stained with Wright-Giemsa. Hematopoietic colony assays were performed on Day 21. 5000 cells were plated in 35 mm plastic culture dishes containing methylcellulose-based medium (Methocult T GF H4435, StemCell Technologies). Cells were incubated for 14 days at 37°C and 5% CO_2_. Colonies were enumerated by microscopy.

The surface marker expression of the cultured erythroid progenitor cells were analyzed by flow cytometry. Cells was harvested on days 7, 14, and 21. All of the conjugated antibodies and the corresponding isotype controls were purchased from BD Pharmingen, Heidelberg, Germany. The antibodies against human cluster of differentiation (CD) molecules we used were CD3-allophycocyanin (APC), CD4-fluorescein isocyanate (FITC), CD8-Phycoerythrin (PE), CD13-FITC, CD14-APC, CD19-PE, CD33-FITC, CD34-FITC, CD38-PE, CD41-FITC, CD45-APC, CD71-FITC, CD117-PE, and glycophorin A (GPA)-PE. Erythroid cells were collected and washed twice in phosphate-buffered saline (PBS) with 0.1% BSA and stained in accordance with the manufacturer's suggested concentration of conjugated antibody for 30 min at 4°C. The stained cells were then washed 2× in PBS + 0.1% BSA and fixed with the wash buffer supplemented with 1% paraformaldehyde. The samples were then analyzed using a flow cytometer (Becton Dickinson, Franklin Lakes, NJ). Cell populations were analyzed with the CellQuest program (Becton Dickinson).

### 2.2. Isolation, Culture, and Characterization of hFLSCs 

hFLSCs were obtained from 24-week human fetal liver tissues as described previously [23]. Human tissue collection for research purpose was approved by Research Ethics Committee of Beijing Institute of Transfusion Medicine. Pregnant women gave written consent for clinical procedure and research use of the embryonic tissues in accordance with the Declaration of Helsinki. hFLSCs were cultured in a medium comprising 45% Dulbecco's modified Eagle's medium (DMEM, Sigma-Aldrich), 45% DMEM/F12 (Sigma-Aldrich), and supplemented with 10% FCS (fetal calf serum; Gibco, Grand Island, NY). Briefly, hFLSCs were prepared using a ceiling culture method. Human fetal liver tissues were minced into 1 mm^3^ pieces and transferred onto human gelatin-coated 25-cm^2^ flasks. 4 mL of hFLSCs culture medium was added onto the ceiling surface, and after 5 h the flasks were turned back over. The tissue pieces were incubated undisturbed for 7 days at 37°C, allowing the hFLSCs to migrate and adhere to the gelatin-coated surface. Once the primary hFLSCs reached confluency, the hFLSCs were digested with 0.25% Trypsin (Sigma-Aldrich) for 5 min at 37°C. After adding hFLSCs medium, cultures were pipetted into single-cell suspensions, centrifuged, resuspended, and replated onto new flasks.

 The surface marker expression of hFLSCs was analyzed by flow cytometry. Stained cells were analysed on a FACS Calibur using Cell Quest software (Becton Dickinson). All of the conjugated antibodies and the corresponding isotype controls were purchased from BD Pharmingen, Heidelberg, Germany. The antibodies we used were CD11b-PE, CD29-PE, CD34-FITC, CD44-FITC, CD45-FITC, CD90-FITC, CD105-PE, and CD144-PE.

### 2.3. Enucleation of Cord Blood-Derived Erythroid Cells *In Vitro*

To induce terminal maturation, proliferating erythroid progenitor cells were cocultured with irradiated (40 Gy) hFLSCs at 2 × 10^6^ cells/mL in StemSpan supplemented with Epo (10 U/mL, Sigma-Aldrich) and iron-saturated human transferrin (400 *μ*g/mL; Sigma-Aldrich). Differentiating erythroblasts were maintained at 2 to 4 × 10^6^ cells/mL for 8 days.

### 2.4. Functional Analysis of Hemoglobin 

Cocultured erythroid cells collected at 8 days were used to characterize the function of hemoglobin. Oxygen equilibrium curves were determined using a Hemox-Analyzer, Model B. The gas phase gradients were obtained using nitrogen and room air, and the curves were run in both directions.

### 2.5. Reverse Transcription-Polymerase Chain Reaction Analysis 

Total RNA was extracted by TRIzol reagent (Invitrogen) according to the manufacturer's instructions. 1 *μ*g RNA was then reverse-transcribed into cDNA by Avian Myeloblastosis Virus (AMV) reverse transcriptase (Takara Bio, Shiga, Japan). PCR was performed with rTaq polymerase (TaKaRa). An aliquot of PCR products was analyzed on 1.5% ethidium bromide-stained agarose. *β*-actin was used as a housekeeping gene to evaluate and compare quality of different cDNA samples. The following primer pairs were used for the amplification of target mRNAs [24]: *ζ* globin (400 base pair (bp)) forward primer 5′-CCA AGA CTG AGA GGA CCA TCA TTG and reverse primer 5′-AGG ACA GAG GATACGACC GATAGG; *ε* globin (212 bp) forward primer 5′-AAG ATG AAT GTG GAA GAG GCT GG and reverse primer 5′-TTA GCAAAG GCG GGC TTG AG; *β*-globin (394 bp) forward primer 5′-GGG CAG GTT GGT ATC AAG GTT AC and reverse primer 5′-GGG GAA AGA AAA CAT CAA GCG; GATA-1 (378 bp) forward primer 5′-TCAATT CAG CAG CCT ATT CC and reverse primer 5′-TTC GAG TCT GAA TAC CAT CC; PU-1 (600 bp) forward primer 5′-CGA CCA TTA CTG GGA CTT CC and reverse primer 5′-TTC TTC TTC ACC TTC TTG ACC; SCL/TAL-1 (356 bp) forward primer 5′-ATG GTG CAG CTG AGT CCT CC and reverse primer 5′-ATA TAC TTC ATG GCC AGG CGG; *β*-actin (222 bp) forward primer 5′-GAT CCA CAT CTG CTG GAA GG and reverse primer 5′-AAG TGT GAC GTT GACATC CG. 

### 2.6. Karyotype Analysis 

Karyotype analyses of hFLSCs were carried out at passages 20 using the standard G-banding procedure. The karyotype analyses were done at the Beijing Institute of Radiation Medicine, Cytogenetics Laboratory (Beijing, China).

## 3. Results

### 3.1. *In Vitro* Large Scale Production of Human Erythroid Progenitors 

**Figure 1:**
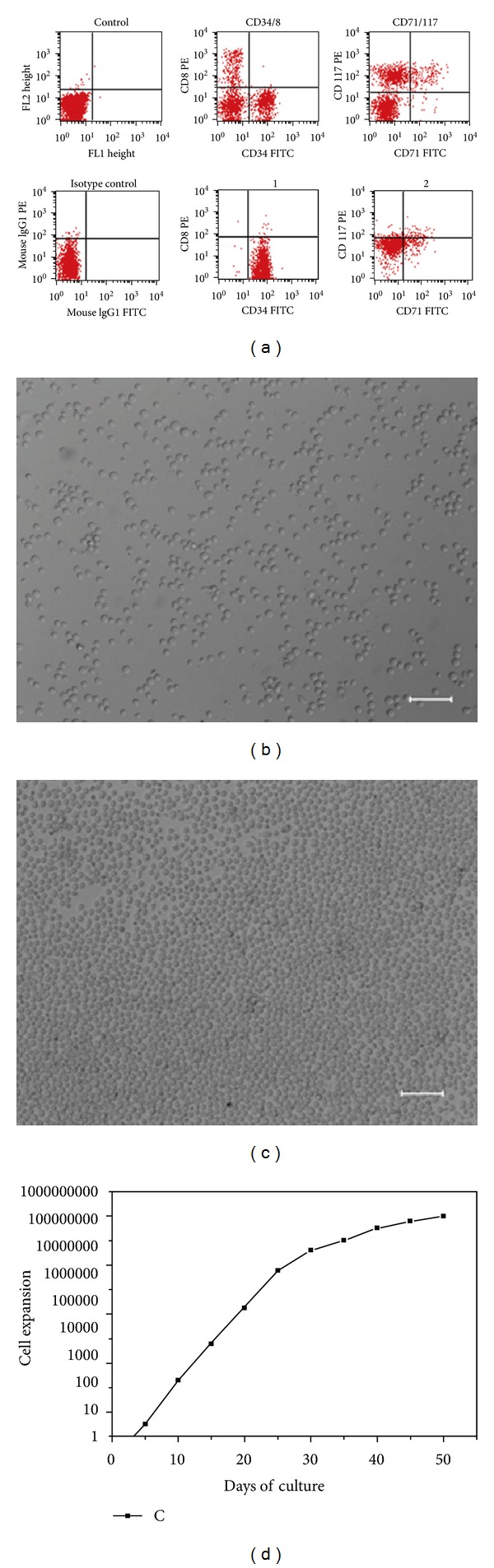
Long-term expansion of human erythroid progenitors from cord blood HSCs. (a) The purity of cord blood derived HSCs was analyzed by flow cytometry. The expression of CD8, CD34, CD71, and CD117 was shown. ((b) and (c)) Morphology of the cultured erythroid progenitor cells in different density. (d) Proliferation kinetics were determined by daily measurements in an electronic cell counter. Cumulative cell numbers were calculated as described in Section 2. Results of one optical conditions are shown.

The purity of the isolated CD34^+^ HSCs was always greater than 95% (Figure 1(a)). CD34^+^ cells derived from cord blood were induced to erythroid progenitors in serum-free medium (StemSpan) by using a combination of Epo, SCF, Dex, and IGF-1. From day 7 on, morphologically homogeneous erythroid progenitors could be expanded into mass cultures in a rapid time (Figures 1(b)-1(c)), which proliferated exponentially for over 50 days, demonstrating a clear capacity for long-term self-renewal (Figure 1(d)). At regular intervals, more mature or apoptotic cells were removed by density gradient centrifugation. After 50 days, the majority of progenitors was undergoing gradual proliferation arrest before eventually undergoing apoptosis. During the entire expansion, an apparently overall increase in cell number of 10^8^-fold to more than 10^9^-fold was obtained (*n* = 5). Under optical conditions, the erythroid cells resulted in a more than 10^9^-fold expansion within 50 days (Figure 1(d)). 

### 3.2. Characterization of Cord Blood-Derived Erythroid Progenitors 

**Figure 2:**
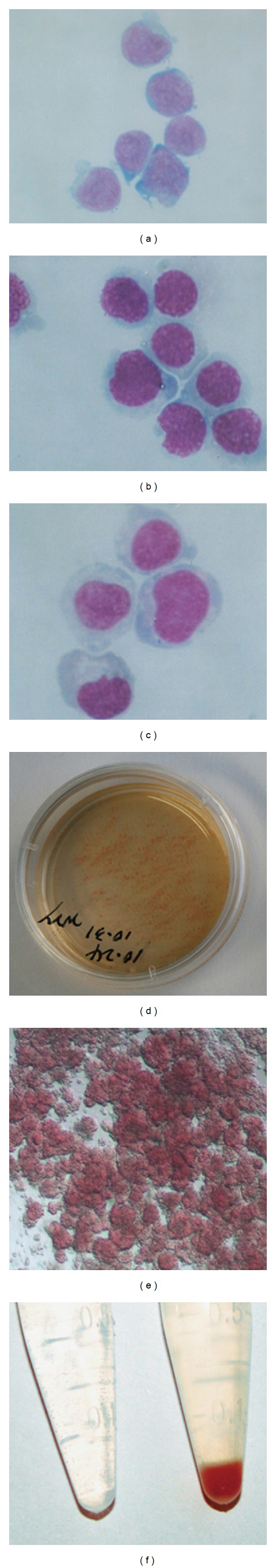
Characterization of cord blood-derived erythroid progenitor cells. ((a)–(c)) Photographs of cytospin preparations of the erythroid progenitor cells stained by Wright-Giemsa. CD34^+^ cells (a), and day 10 (b) and day 15 (c) erythroid progenitors were cytospined on a polylysine-coated slide and stained with Wright-Giemsa reagents. Original magnification: ×400. ((d) and (e)) Methyl-cellulose assays of 21 days erythroid cells. (f) Erythroid progenitor cells (pellet) cultured for 21 days *in vitro*. Left is PBS as control.

Morphologically, the erythroid progenitors obtained using the above protocol were nucleated and substantially larger than definitive erythocytes with an average diameter of approximately 10 *μ*m. Giemsa-Wright staining showed the morphology of the cultured erythroid progenitor cells (Figures 2(a)–2(c)). In order to characterize the morphological changes of the cultured cells through the stages of erythropoiesis, cell morphology was assessed by microscopic examination, at day 14, greater than 95% of the cells in culture were erythroid cells, and less than 5% of cells morphologically appeared to be promyelocytes and monocytes. 

Our protocol for proliferative induction only targeted erythroid progenitors, namely, Burst Forming Unit-Erythroid (BFU-E), Colony Forming Unit-Erythroid (CFU-E) but not granulomacrophagic progenitors, namely Colony Forming Unit Granulomacrophagic (CFU-GM) (Figures 2(d) and 2(e)). Hematopoietic colonies formation assay showed that most induced cells of our system are erythroid progenitors. 

### 3.3. Flow Cytometric Analysis of Differentiation into Erythroid Progenitor Cells

**Figure 3:**
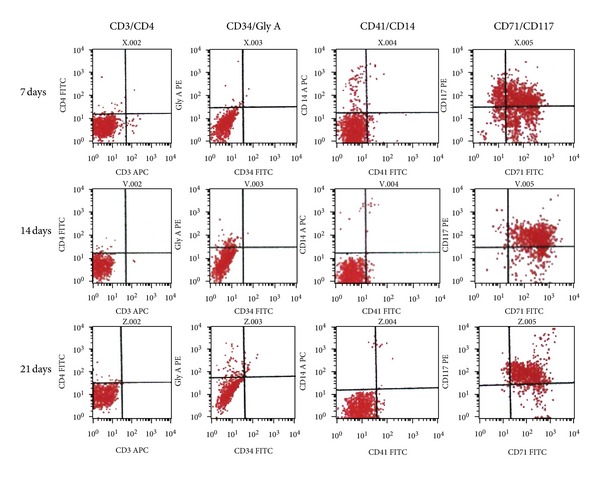
Progression of cell-surface marker expression in developing erythroid progenitor cell cultures. Aliquots of erythroid cells were harvested at the times indicated, stained with combinations of fluorescently labeled antibodies against markers characteristic for different hematopoietic lineages and stages of development, and subjected to flow cytometry.

Differentiation over time of CD34^+^ cells was documented by FACS analysis of cell surface markers (Figure 3 and Table 1). At day 21, 95% were CD71 positive, 90% were CD117 positive, and 15% of the cells expressed GPA, whereas the majority of the cells did not express myelomonocytic or megakaryocytic or lymphatic antigens (1% of cells expressed CD14; 2.4% of cells expressed CD41; 3% of cells expressed CD4; 2.5% of cells expressed CD8) and progenitor antigens (0.7% cells were positive for CD34; 0.5% cells expressed CD38) (Table 1).

CD34^+^ cells rapidly declined, to be quite absent on day 14 (Figure 3). This is expected since CD34 is a marker of early progenitor cells and, in the erythroid lineage, is not expressed after the BFU-E stages. In contrast, GPA, which is a marker of more mature erythroid cells, first appears at the basophilic erythroblast stage and its expression increases throughout the rest of erythroid differentiation [16]. As expected, GPA expression increased almost linearly by the culture time. 

### 3.4. Isolation and Characterization of hFLSCs

**Figure 4:**
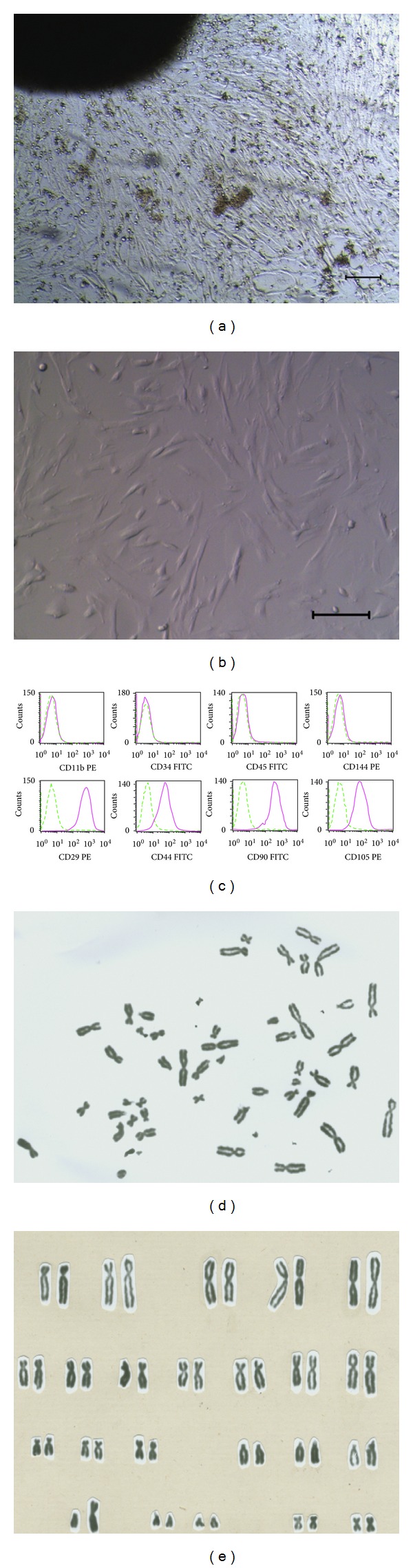
Isolation and characterization of human fetal liver stromal cells (hFLSCs). ((a) and (b)): hFLSCs derived from 24-week fetal liver showed morphology of flattened fibroblast-like cells when grown to confluency. Bars = 100 *μ*m. (c) Surface marker of hFLSCs analyzed by flow cytometry. ((d) and (e)) Normal 46, XY karyotype of hFLSCs for 20 passages.

Because fetal liver is an important hematopoietic organ for erythroid development, we hypothesized that hFLSCs might produce protein(s) that support *in vitro* terminal maturation of erythroid progenitors. We isolated and cultured hFLSCs from 24-week human fetal liver. After one week culture, the stromal cells migrated out the border of the tissue clumps (Figures 4(a) and 4(b)), and it took two weeks for the cells to become confluent. After the primary and three subsequent passages, hFLSCs were adherent to be fusiform shape and highly uniform in morphology (Figure 4(a)). As hFLSCs are propagated in culture for more than 30 passages, there are no obviously cell morphology changes. Flow cytometry analysis was used to characterize cell surface markers of hFLSCs, showing that the hFLSCs were positive for some stromal progenitor markers, such as CD29, CD44, CD71, CD90, and CD105, but negative for CD34 and CD45 (Figure 4(c)). hFLSCs maintained normal karyotypes after 20 passages (Figures 4(d) and 4(e)).

### 3.5. Enucleation and Maturation of Cord Blood-Derived Erythroid Cells *In Vitro*

**Figure 5:**
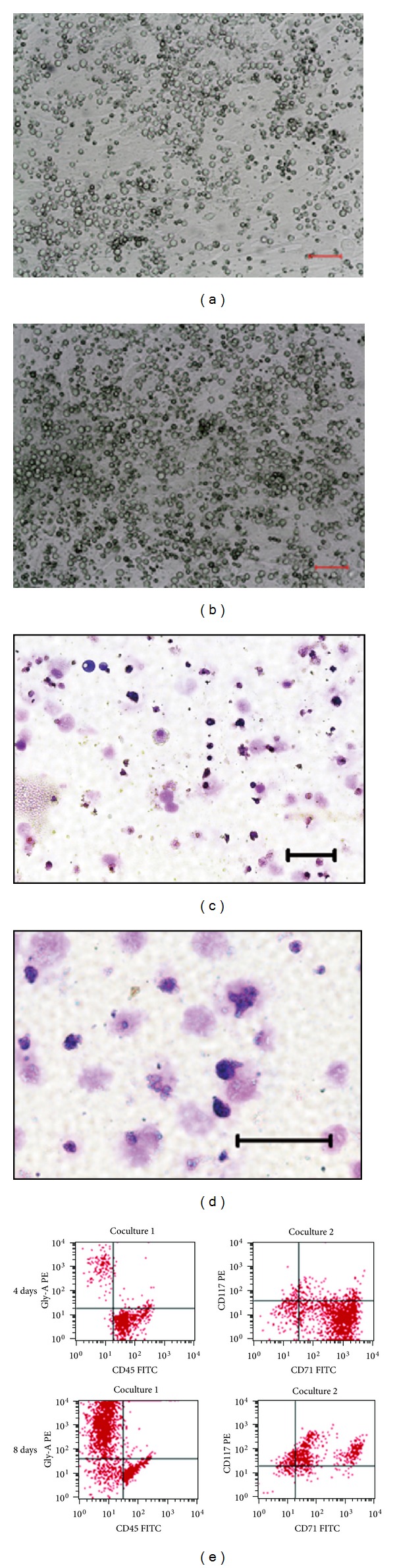
Enucleation of cord blood HSCs-derived erythroid cells *in vitro*. ((a) and (b)) Erythroid progenitors cocultured with hFLSCs for 4 days (a) and 8 days (b). Bars = 100 *μ*m. ((c) and (d)) Erythroid cells derived from human cord blood HSCs were cocultured with hFLSCs for 8 days. On day 4 (c) and 8 (d), cells were cytospun and stained with Wright-Giemsa dye. Bars = 100 *μ*m. (e) Maturation of cord blood-derived erythroid cells mimic erythroid development. Expression of GPA, a mature erythrocyte marker, increases with time and CD71, an immature red blood cell marker, shows a decrease in expression over time.

A critical scientific and clinical issue is over whether cultured erythroid cells can be matured *in vitro* to generate enucleated erythrocytes. We tried to induce erythroid progenitors to terminal maturation by coculturing with hFLSCs. Erythroid progenitor cells cultured in this condition with hFLSCs to induce terminal maturation. And the cells expanded one or more times when cocultured with hFLSCs (Figures 5(a) and 5(b)). Approximately 60–80% of erythroid cells were enucleated when these cells were transferred to hFLSCs layers from nonstromal cultures and cocultured from days 8 (Figures 5(c) and 5(d)). Erythroid progenitor cells kept in liquid conditions (without transfer to hFLSCs) could enucleate 10%–30% suggest that enucleation could not be achieved completely in feeder-free condition. 

To further investigate the events associated with enucleation, we examined cell surface marker expression related to the process of erythrocyte maturation. We observed a progressive decrease of CD117, an early erythroblast marker, on day 8 and decreased their expression over time. And they showed low to negligible level of GPA protein, a mature erythrocyte marker, at the beginning, but increased their expression dramatically with their maturation (Figure 5(e)).

### 3.6. Gene Expression during Erythropoiesis

**Figure 6:**
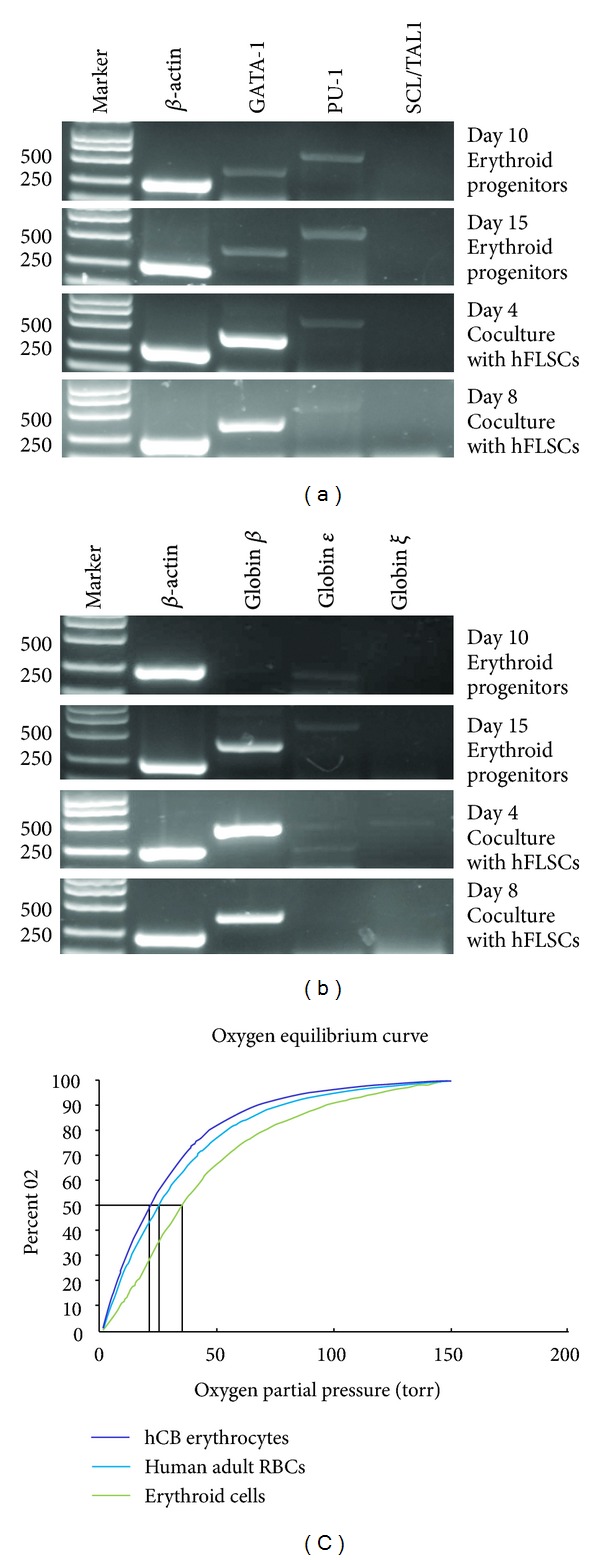
Characterization of cord blood HSCs-derived red blood cells. (a) RT-PCR analysis for erythroid specific transcriptor gene expression. (b) RT-PCR analysis for globin gene expression. Gene expression was normalized to the internal control, *β*-actin gene, for each data point. (c) Functional characterization of cord blood HSCs-derived erythroid cells. Oxygen equilibrium curves of normal human adult and cord blood RBCs and cord blood-derived RBCs by coculturing with hFLSCs for 8 days.

 Erythroid progenitors cultured at day 10 and 15 and enucleated cells at day 4 and 8 were analyzed for erythroid gene expression (*ζ*, *ε*, and *β* globins, GATA1, PU-1, and SCL/TAL-1) (Figures 6(a) and 6(b)). The expression level of transcription factor GATA1 mRNA was upregulated during the early stages of erythroid differentiation and then downregulated when cocultured with hFLSCs. At an early stage of differentiation, *ζ* and *ε*-globin mRNA was hardly expressed; during maturation the expression level of *β*-globin mRNA increased. At cocultured day 8, the enucleated cells only expressed the *β*-globin.

### 3.7. Oxygen Equilibrium Analysis

The oxygen equilibrium curves of the cord blood-derived erythroid cells (cultured with hFLSCs for 8 days) were either very similar to or somewhat rightward shifted (Figure 6(c)), relative to those of human cord blood and normal adult RBCs. 

## 4. Discussion

The expansion of hematopoietic progenitors from umbilical cord blood *ex vivo* is of great interest, both for basic research and the exploitation of clinical potential. This work described a method which enabled the purification and *in vitro* expansion of large numbers of erythroid progenitors from cord blood-derived HSCs. This system could be used to reproducibly generate large numbers of erythroid cells under serum-free conditions. 

In the presence of Epo, SCF, IGF-1, and Dex added after 2 week of culture, almost all the cells were erythroid progenitor cells positive to erythroid specific cell surface markers. After long time culture, the culture conditions resulted in a great cell expansion and accumulation of erythroid cells. So, Epo, SCF, IGF-1, and Dex represented the most potent factors required for inducing proliferation of erythroid progenitor cell [1, 20, 21, 25]. The complete erythroid differentiation and maturation were documented by the morphological analysis and by changes in surface markers expression. More than 90% of the cells expressed GPA after 8 days of being cocultured with hFLSCs. 

CD71 is highly expressed on both proliferating cells of various cell types and early erythroid cells with high iron uptake for Hb synthesis [26, 27]. After day 14 in the erythroid progenitor cells expansion conditions, CD71 expression is up to 97%. And on the last culture days with hFLSCs almost all cells expressed GPA. Furthermore, most GPA^+^cells became CD71^−^, which is known as a sign of cell cycle arrest and terminal maturation to erythrocytes [28].

Previously used procedures failed to yield long-term proliferating but immature erythroid progenitors, due to massive spontaneous differentiation [12, 29]. Using serum-free media together with Epo, SCF, IGF-1, and Dex we could expand human erythroid progenitors from cord blood derived HSCs for more than 50 days. This yielded mass populations with an *in vitro* lifespan of approximately 30–35 generations, almost absent from spontaneous differentiation largely facilitating future biochemical and molecular characterization. Importantly, optimization of differentiation conditions allowed induction of synchronous terminal maturation, resulting in enucleated cells virtually indistinguishable from erythrocytes of peripheral blood. 

In our hands, the serum-free medium used here was critical for generating long-term cultures. Other serum-free media proved to be less efficient. Compared with other current procedures, it should also be noted that our methods did not require negative selection (depletion) against lymphoid or granulocytic cells [30]. Routinely, a (calculated) 10^8^–10^9^-fold erythroid cells expansion was obtained, in some cases even a (calculated) 10^10^-fold increase was obtained (about 35 generations). Thus, the procedure described here enables the production of large numbers of homogeneous primary erythroid cells for detailed molecular characterization of mechanisms.

*In vivo*, stromal cells form a complex microenvironment for HSCs that controls their multiple fates, including apoptosis, migration, and the cell divisions that lead to formation of lineage-committed progenitors [31, 32]. Different types of stromal and other cell types can have either positive or negative effects on fates of HSCs. In previous studies, terminal differentiation and enucleation of erythrocytes *in vitro* were reported to be dependent on macrophages presence in cultures [33, 34]. Giarratana et al. [13, 35] recently described a protocol to achieve *in vitro* terminal erythroid maturation (90%–100% of enucleated erythrocytes) using the murine stromal cell lines MS5 or human stromal cell lines to reproduce *ex vivo* the microenvironment existing in vivo. In our model, terminal erythroid maturation was satisfactorily achieved in coculturing with hFLSCs, making the culture system more safe. 

However, the underlining mechanism of the hFLSCs during erythroid differentiation was not fully explained in the present study. There are some other studies showed that the mouse and human FLSCs can promote the *in vitro* generation of erythroid cells from different sources of stem cells [36–38]. All these studies suggested that the fetal liver stromal cells might mimic the hematopoietic supportive environment of the fetal liver *in vitro* through many different pathways [39]. 

Erythropoiesis at molecular level is driven by a combination of transacting factors that act in concert to direct the genes expression for erythroid-specific proteins [40, 41]. We established that culture conditions of cord blood-derived HSCs allow to reproduce normal erythroid differentiation confirmed also at the molecular level. Genes required for primitive erythropoiesis were expressed at high levels in early days of cultures. Globin gene expression in the maturing erythrocytes *in vitro* recapitulated expression patterns which occur in adult hemoglobin expression well [42, 43]. A more recent study has used the *in vitro* culture model to study the molecular mechanisms of erythropoiesis. The results show that KLF1 is a critical activator of the BCL11A gene, encoding a suppressor of HbF expression [44]. And, little has been known so far about enucleation mechanism of erythroblasts. Our methods also provided a new model to investigate molecular and cellular mechanisms involved in the enucleation of human RBCs.

We demonstrated here that erythroid cells derived from cord blood HSCs possessed oxygen equilibrium curves comparable to normal cord blood and adult RBCs. This finding demonstrated that the cord blood-derived RBCs have oxygen carrying properties that are comparable to those of normal adult erythrocytes. Another critical issue for clinical utilization of cord blood-derived RBCs is about whether they can be enucleated *in vitro*. hFLSCs coculture improved efficiency of enucleation, but even without these cells enucleation could not be achieved for a completely feeder-free system. We showed that 60%–80% of the RBCs underwent multiple differentiation events, including a progressive decrease in size and increase in GPA expression (a mature RBC marker), which is similar to normal RBCs. However, further study will be necessary to investigate cord blood-derived erythroid cells in vivo function.

Taken together, culture method described here could facilitate extensive biochemical analyses of erythropoiesis, which were technically demanding so far due to lack of material. Our system also provided a useful basis for future production of available RBCs for transfusion. 

## Figures and Tables

**Table 1:** Flow cytometry analysis of cell surface markers during erythroid progenitor cells differentiation from cord blood derived HSCs (mean ± SD of triplicate assays).

Marker	7 day (%)	14 day (%)	21 day (%)
CD3*	1.78 ± 0.23	0.14 ± 0.08	0.42 ± 0.26
CD8*	1.14 ± 0.19	1.76 ± 0.40	2.31 ± 0.98
CD14*	6.20 ± 1.48	1.4 ± 0.29	2.11 ± 1.20
CD33*	81.23 ± 2.17	66.02 ± 1.93	59.41 ± 4.08
CD38*	30.65 ± 4.42	3.28 ± 0.85	0.84 ± 0.31
CD45	97.15 ± 1.43	96.28 ± 1.78	96.31 ± 0.80
GPA*	1.15 ± 0.51	7.49 ± 1.32	14.92 ± 2.12
CD4*	1.39 ± 0.28	1.16 ± 0.59	3.60 ± 1.27
CD13*	26.40 ± 1.86	4.72 ± 1.51	8.20 ± 1.26
CD19	1.21 ± 0.22	0.08 ± 0.04	0.04 ± 0.03
CD34	0.53 ± 0.11	0.50 ± 0.41	0.64 ± 0.25
CD41*	4.24 ± 1.21	3.18 ± 0.76	2.55 ± 1.57
CD71*	73.46 ± 2.89	96.32 ± 1.44	94.44 ± 0.67
CD117*	63.56 ± 7.05	83.57 ± 3.13	88.72 ± 2.63

The cell surface markers expression of cultured erythroid progenitor cells significantly changed. The expression of CD4, CD8, CD71, CD117, and GPA significantly increased, while the CD3, CD13, CD14, CD33, CD38, and CD41 significantly decreased (*P value < 0.01, using Student's *t*-test).
